# Sgo1 Regulates Both Condensin and Ipl1/Aurora B to Promote Chromosome Biorientation

**DOI:** 10.1371/journal.pgen.1004411

**Published:** 2014-06-19

**Authors:** Karolina Peplowska, Andreas U. Wallek, Zuzana Storchova

**Affiliations:** Group Maintenance of Genome Stability, Max Planck Institute of Biochemistry, Martinsried, Germany; University of Tokyo, United States of America

## Abstract

Correct chromosome segregation is essential in order to prevent aneuploidy. To segregate sister chromatids equally to daughter cells, the sisters must attach to microtubules emanating from opposite spindle poles. This so-called biorientation manifests itself by increased tension and conformational changes across kinetochores and pericentric chromatin. Tensionless attachments are dissolved by the activity of the conserved mitotic kinase Aurora B/Ipl1, thereby promoting the formation of correctly attached chromosomes. Recruitment of the conserved centromeric protein shugoshin is essential for biorientation, but its exact role has been enigmatic. Here, we identify a novel function of shugoshin (Sgo1 in budding yeast) that together with the protein phosphatase PP2A-Rts1 ensures localization of condensin to the centromeric chromatin in yeast *Saccharomyces cerevisiae*. Failure to recruit condensin results in an abnormal conformation of the pericentric region and impairs the correction of tensionless chromosome attachments. Moreover, we found that shugoshin is required for maintaining Aurora B/Ipl1 localization on kinetochores during metaphase. Thus, shugoshin has a dual function in promoting biorientation in budding yeast: first, by its ability to facilitate condensin recruitment it modulates the conformation of the pericentric chromatin. Second, shugoshin contributes to the maintenance of Aurora B/Ipl1 at the kinetochore during gradual establishment of bipolarity in budding yeast mitosis. Our findings identify shugoshin as a versatile molecular adaptor that governs chromosome biorientation.

## Author Summary

Accurate chromosome segregation is required for the equal distribution of genetic information to progeny. Failure to equally segregate chromosomes leads to aneuploidy, cell death or cancer. Proteins of the conserved shugoshin family contribute to accurate chromosome segregation in both meiosis and mitosis. The role of shugoshin in protection of centromeric cohesion during meiosis is well understood, but only little is known about shugoshin's function during mitosis. We show that Sgo1 mediates localization of the heterotrimeric phosphatase PP2A-Rts1 to the centromere and that this is in turn important for the efficient recruitment of condensin to the centromere. The failure to load centromeric condensin results in a defect during correction of improper microtubule-kinetochore attachments. Moreover, Sgo1 facilitates the maintenance of a centromeric pool of Aurora B/Ipl1, a conserved mitotic kinase essential for the correction of faulty microtubule-kinetochore attachments. Our results show that Sgo1 operates as a multifunctional hub that coordinates two centromeric functions essential for correct chromosome segregation.

## Introduction

Accurate chromosome segregation into daughter cells requires the formation of correct chromosome attachments to the mitotic spindle. Each of the sister kinetochores has to attach to microtubules (MTs) emanating from opposite spindle poles in order to achieve biorientation of sister chromatids. Correctly attached and bioriented kinetochores (KTs) generate tension caused by attached MTs, which is, in turn, opposed by sister chromatid cohesion [1]. Consequently, biorientation is achieved by stabilization of correct bipolar attachments that generate tension and continuous dissolution of incorrect, tensionless attachments [2], [3]. The spindle assembly checkpoint (SAC) recognizes KTs lacking attachments and halts the progression from metaphase to anaphase until all pairs of sister chromatids become bioriented. How exactly cells recognize erroneous tensionless attachments is not well understood. The chromosome passenger complex (CPC) consisting of the conserved kinase Aurora B/Ipl1, Incenp/Sli15, Survivin/Bir1, and Borealin/Nbl1 plays a crucial role in dissolving faulty connections by phosphorylating multiple substrates at the MT-KT interface, thereby creating unattached KTs leading, in turn, to SAC activation (reviewed in [1]). Other proteins, most prominently the conserved shugoshin/MEI-S322 family of proteins, have been proposed to facilitate the establishment of biorientation by promoting the correction of tensionless attachments [4]–[7]. Shugoshins recruit the heterotrimeric protein phosphatase PP2A-B56/Rts1 to the centromere via their conserved N-terminal coiled-coil domain to ensure the protection of cohesion during meiosis and in many organisms also in mitosis [8]–[11]. Besides this canonical function, shugoshin also affects Aurora B/Ipl1 kinase in mitosis by facilitating its centromeric localization in fission yeast [5], [12]. Moreover, Aurora B kinase activity in *Xenopus* egg extracts is impaired upon depletion of Sgo2 [13]. However, the exact function of shugoshin and PP2A in tension sensing and the establishment of biorientation remains unclear.

Kinetochores, large proteinaceous complexes assembled on centromeric DNA, are crucial interaction hubs for events necessary for accurate chromosome segregation. KTs are flexible and dynamic structures that become stretched when MTs attach and bipolarity is achieved [14], [15]. Furthermore, centromeric and pericentric chromatin creates a flexible spring-like filament that is responsive to the tension exerted by MT-mediated pulling forces of the spindle [16], [17]. This elasticity is ensured by the concerted activity of several multi-protein complexes including cohesin and condensin that bind and organize the pericentric chromatin into inter- and intramolecular loops (reviewed in [18]). Interestingly, mutants that are not able to maintain the functional organization of the pericentric region fail to create bioriented MT-KT attachments during mitosis [19]–[21]. Cohesin has been shown to control KT geometry [22] and condensin in *C. elegans* is required for centromere resolution [23]. Moreover, depletion of condensin I suppresses KT stretching in HeLa cells, thereby causing a SAC-mediated mitotic delay [15]. Taken together, cohesin and condensin contribute to the architecture and elastic properties of pericentric chromatin. However, whether the pericentric architecture modulates CPC activity or localization in response to lack of tension on sister KTs and how it affects the turnover of erroneous MT-KT attachments has not been clarified so far.

Here, we show that Sgo1 function is essential for the recruitment of condensin to the pericentric region. Moreover, Sgo1 localizes the PP2A subunit Rts1 to the same region, which facilitates the binding of condensin to centromeric chromatin. The failure to load functional condensin onto pericentric chromatin impairs the cellular response to tensionless attachments. Additionally, Sgo1 is required for the maintenance of the Ipl1 kinase on centromeres, which, in turn, allows for correction of erroneous MT-KT attachments. We propose that Sgo1 is a scaffold protein that via spatio-temporal modulation of kinase and phosphatase activity on KTs ensures a dual function - organization of the pericentric chromatin that mediates tension sensing and the maintenance of centromeric Ipl1 that facilitates correction of tensionless attachments.

## Results

### Sgo1-mediated recruitment of PP2A-Rts1 to the centromere is required for biorientation

**Figure 1:**
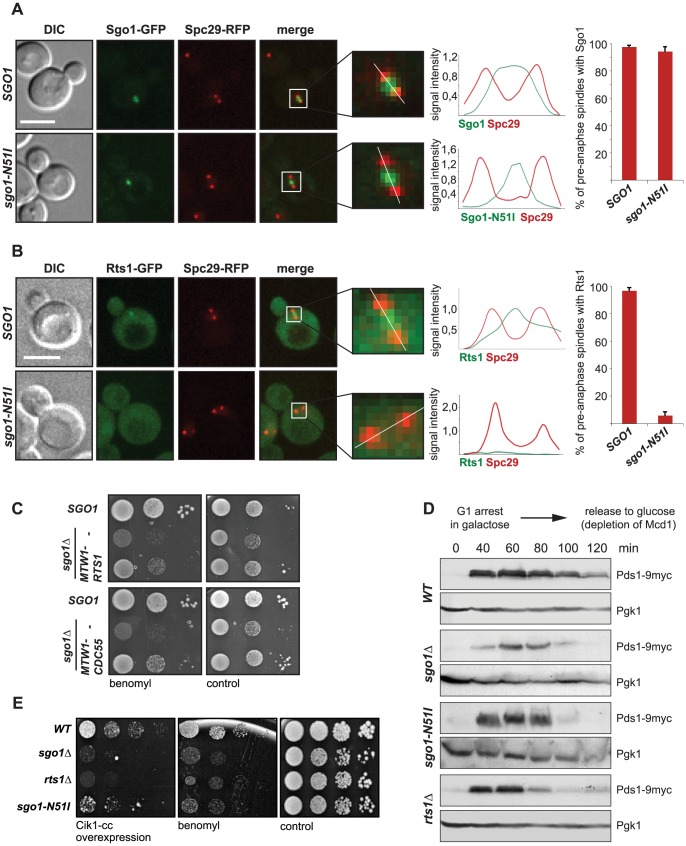
Sgo1-mediated PP2A recruitment to the centromere is essential for tension sensing. (A) Localization of the GFP-tagged Sgo1 and Sgo1-N51I mutant. Spindle pole bodies (SPBs) are visualized with Spc29-RFP. Only pre-anaphase spindles (SPB distance <2 µm, spindle located in the mother cell) were scored. Plots on the right: histogram of signal intensity across the white line in the insets and percentage of cells with localized GFP signal. Bar – 5 µm. (B) Localization of Rts1-GFP in wild type cells or in cells carrying the *sgo1-N51I* mutation. SPBs are visualized with Spc29-RFP. Only pre-anaphase spindles (SPB distance <2 µm, spindle located in the mother cell) were scored. Plots on the right: histogram of signal intensity across the white line in the insets and percentage of cells with localized GFP signal. Bar – 5 µm. (C) Sensitivity to the microtubule depolymerizing drug benomyl in cells lacking *SGO1*. Cell viability was scored upon artificial tethering of the PP2A regulatory subunit Rts1 and Cdc55, respectively, to the kinetochore. (D) Progression of the wild type, *sgo1Δ, sgo1-N51I* and *rts1Δ* mutants through cell cycle upon depletion of the cohesin subunit Mcd1 which leads to formation of tensionless kinetochores. (E) Sensitivity of the wild type, *sgo1Δ, rts1Δ* and *sgo1-N51I* mutants to microtubule poisons and to the overexpression of Cik1-cc which triggers the formation of syntelic attachments at high frequencies.

Shugoshin proteins interact with PP2A-B56/Rts1 via their conserved N-terminal coiled-coil domains [8]–[11]. Earlier studies in higher eukaryotes and budding yeast have shown that substitution of a highly conserved asparagine residue within the N-terminus of Sgo1 abolishes the interaction with PP2A while retaining other functions of the protein [11]. We introduced this substitution (N51I) into budding yeast Sgo1 in order to study the role of the Sgo1-PP2A interaction during mitosis. As expected, the N51I mutation abrogated the interaction between the recombinantly purified N-terminus of Sgo1 and PP2A-Rts1 complexes purified from yeast mitotic lysates (Figure S1A), but did not affect the proper localization of the mutant protein to the centromeric region during mitosis (Figure 1A). PP2A is a ubiquitously expressed serine/threonine phosphatase with a broad substrate specificity [24]. It consists of a dimeric catalytic core that interacts with various regulatory subunits to promote diverse cellular functions. We found that the binding to Sgo1 is specific for PP2A complexes containing the Rts1 regulatory subunit, as we did not observe any specific interaction between Sgo1 and PP2A containing the regulatory subunit B55/Cdc55 (Figure S1B). Rts1 localizes diffusely to the cytoplasm and nucleus, but it becomes enriched specifically at the centromeric region in pre-anaphase cells (Figure 1B, [25]). Replacement of wild type *SGO1* with the *sgo1*-*N51I* allele resulted in the reduction of the Rts1-GFP signal intensity between the spindle pole bodies (SPBs) (Figure 1B). Chromatin immunoprecipitation (ChIP) of Rts1-FLAG confirmed that, in comparison to a control locus, Rts1 is enriched at centromeres and this enrichment depends on the presence of Sgo1 in mitotic cells (Figure S1C), similarly as previously shown in budding yeast meiosis [26]. To further elucidate the interplay between Sgo1 and the two forms of PP2A, we analyzed genetic interactions among respective mutants. We found that the *sgo1*Δ *rts1*Δ double mutant shows a comparable growth defect as *sgo1*Δ alone and the *sgo1-N51I rts1*Δ mutant has a milder phenotype resembling *rts1*Δ. In contrast, we observed an additive growth defect of the *sgo1*Δ *cdc55*Δ double deletion that was only slightly improved by introduction of the *sgo1-N51I* allele (Figure S1D). Thus, protein interaction and localization analyses as well as genetic evidence suggest that Sgo1 interacts with PP2A-Rts1 (and not with PP2A-Cdc55) via its N-terminal coiled-coil domain.

Loss of Sgo1 or introduction of the *sgo1-N51I* mutation causes sensitivity to the microtubule-destabilizing drug benomyl, indicating that the recruitment of PP2A-Rts1 is crucial for Sgo1's functions in mitosis (Figure 1E, S2A, B). Importantly, this sensitivity was alleviated by artificially tethering Rts1 to the KT via fusion to the inner kinetochore protein Mtw1 (Figure 1C). Remarkably, artificial tethering of the second PP2A regulatory subunit Cdc55 partially rescued the benomyl sensitivity of *sgo1*Δ cells as well (Figure 1C), whereas the control Mtw1-GFP fusion protein did not affect the benomyl sensitivity of *sgo1*Δ cells (Figure S2C, D). Also, a fusion protein of mutant Sgo1-N51I and Cdc55 suppressed the sensitivity of *sgo1*Δ cells (Figure S2E). The observed rescuing effects cannot be explained by increased levels of PP2A regulatory subunits, because massive overexpression of Rts1 or Cdc55 by itself did not restore the growth of *sgo1*Δ cells on plates containing benomyl (Figure S2F, G). These observations suggest that the localization of PP2A complexes at the centromere is required for correct chromosome segregation in the presence of microtubule poisons.

By visualizing TetO arrays integrated 1 kb away from the centromere on chromosome 4 [27], we determined that loss of the centromeric Rts1 pool increases the frequency of segregation errors (Figure S3A). We found that 86% of wild type cells segregated chromosome 4 correctly into the daughter cells within 75 min after release from nocodazole arrest which elevates the rates of syntelic, tensionless MT-KT attachments (Figure S3A, B). In contrast, only about 50% of cells expressing the Sgo1-N51I mutant correctly segregated chromosome 4 after release from nocodazole-induced cell cycle arrest (Figure S3A, B). Similar results were obtained with cells lacking Rts1 (Figure S3A, B). The elevated frequency of chromosome missegregation in *rts1Δ* strains was rescued to the wild type level upon genetic complementation with a vector carrying *RTS1* under the control of its endogenous promoter (Figure S3B). This finding supports the notion that Sgo1-dependent Rts1 localization to centromeres is important for the correction of erroneous MT-KT attachments.

The lack of Sgo1 or Rts1 leads to a failure to halt cell cycle progression upon loss of functional sister chromatid cohesion resulting in tensionless MT-KT attachments (Figure 1D, [4], [11]). In contrast, the SAC response in the presence of the microtubule depolymerizing drug nocodazole that creates unattached KTs is fully preserved (Figure S3C, [4]). To further elaborate the role of Sgo1/PP2A-Rts1 in the repair of tensionless MT-KT attachments, we took advantage of a recently developed genetic tool that promotes the formation of syntelic attachments at high frequencies by overexpression of the coiled-coil domain (Cik1-cc; amino acids 81-360) of the kinesin co-factor Cik1 [28]. Importantly, overexpression of Cik1-cc does not impair spindle geometry or localization of kinetochore proteins [28]. Galactose-induced overexpression of Cik1-cc is lethal in cells lacking Sgo1 (Figure 1E, [28]). The presence of Rts1 on centromeres also contributes to correct chromosome segregation, because cells lacking the Sgo1-Rts1 interaction or Rts1 alone cannot proliferate in conditions in which syntelic attachments are created at high frequencies (Figure 1E). Similarly, both *rts1*Δ and the *sgo1-N51I* mutant showed increased sensitivity to microtubule depolymerizing drugs (Figure 1E, S2A). Taken together, our results show that both Sgo1 and PP2A activities at the centromere are important for accurate chromosome segregation and play a critical role in inducing a cell cycle delay upon formation of tensionless, syntelic attachments. Yet, the finding that the *sgo1*Δ strain shows a stronger phenotype than *rts1*Δ and *sgo1-N51I* shows that Sgo1 performs both Rts1-dependent and Rts1-independent functions during chromosome segregation.

### Lack of functional Sgo1 and Rts1 causes loss of condensin from centromeres

**Figure 2:**
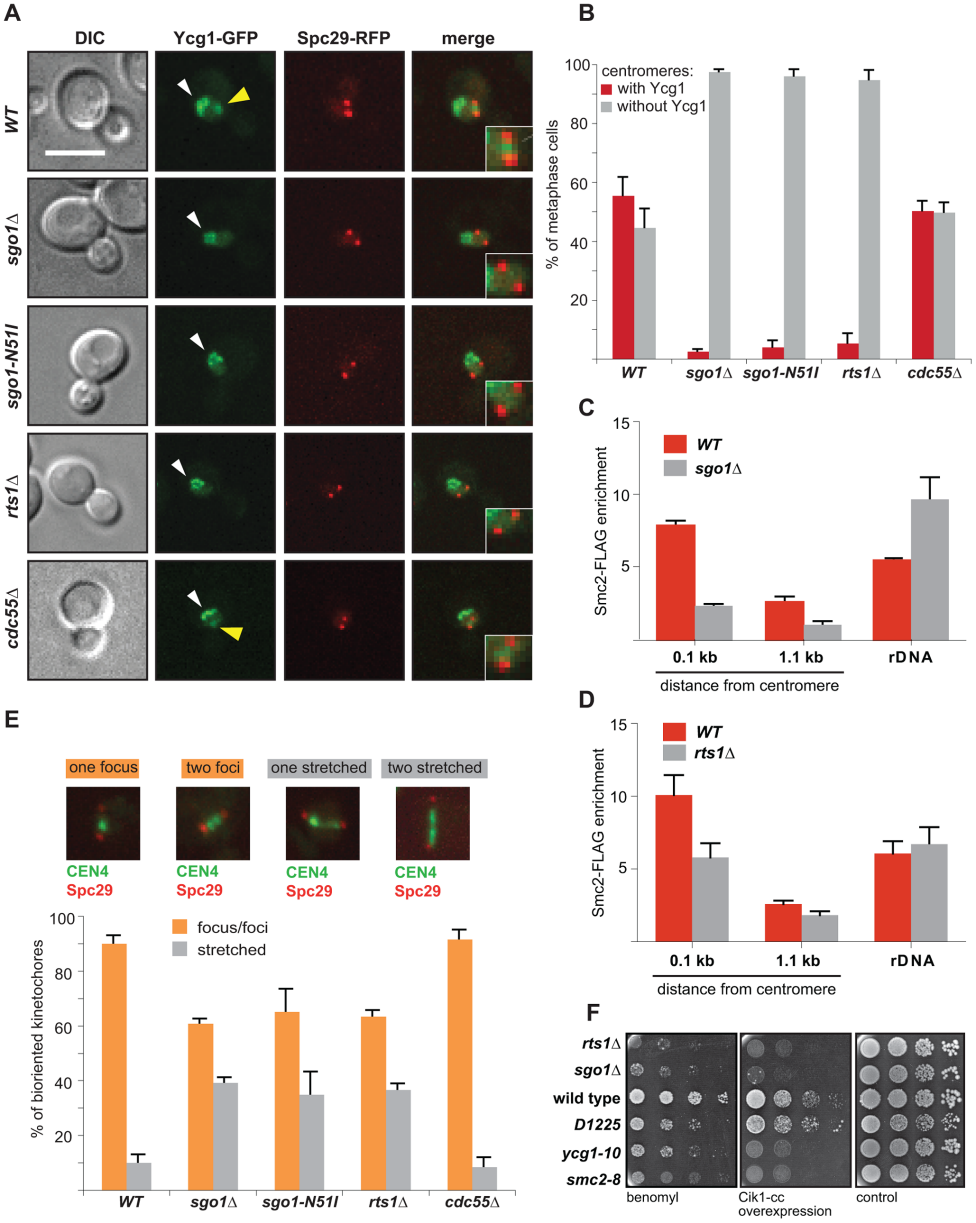
Sgo1 and Rts1 are essential for the maintenance of condensin at centromeres. (A) Localization of the condensin subunit Ycg1-GFP in wild type cells and cells lacking Sgo1, centromeric Rts1 (*rts1Δ* and *sgo1-N51I*) or Cdc55. SPBs are visualized with Spc29-RFP. Bar – 5 µm. Yellow arrowhead: centromeric Ycg1-GFP signal; white arrowhead: Ycg1-GFP on rDNA. (B) Quantification of Ycg1-GFP localization. Only pre-anaphase spindles (SPB distance <2 µm, spindle located in the mother cell) were scored. Means with SD of three independent experiments are shown. At least 150 cells were scored in each experiment. (C) Enrichment of Smc2-FLAG on centromeric DNA (0.1 kb away from CEN1 and 1.1 kb away from CEN4) and on rDNA (NTS1-2) normalized to the levels of Smc2-FLAG bound to the arm of chromosome 10 in mitotic cells. Chromatin immunoprecipitation(ChIP)-qPCR experiments of Smc2-FLAG were performed using wild type and *sgo1*Δ cells arrested with nocodazole. Error bars represent the standard error of the mean. (D) Enrichment of Smc2-FLAG on centromeric DNA (0.1 kb away from CEN1 and 1.1 kb away from CEN4) and on rDNA (NTS1-2) normalized to the levels of Smc2-FLAG bound to the arm of chromosome 10 in mitotic cells. ChIP-qPCR experiments of Smc2-FLAG were performed using wild type and *rts1*Δ cells arrested with nocodazole. Error bars represent the standard error of the mean. (E) Stretching of centromeric DNA in wild type cells compared with cells that fail to localize Rts1 to the centromeric region (*rts1*Δ and *sgo1-N51I*) and with cells lacking Cdc55. CEN4 is visualized by TetR-GFP recruited to the TetO-repeats integrated 1 kb from the centromere. Only pre-anaphase spindles (SPB distance <2 µm, spindle located in the mother cell) were scored. Top – examples of scored categories. (F) Sensitivity of wild type cells and the indicated condensin mutants to microtubule poisons and to the overexpression of the Cik1-cc construct which triggers formation of syntelic attachments at high frequencies. Yeast strain D1225 expresses the non-posphorylatable mutants of the condensin subunits Ycg1, Brn1, and Ycs4 which impair anaphase-specific functions of condensin.

The response to tension across sister KTs is mediated by the structural integrity of pericentric chromatin that is maintained by cohesin and condensin complexes [16], [29], [30]. Therefore, we speculated that the defect observed in *sgo1*Δ and *rts1*Δ cells might result from an altered centromeric architecture. Since localization of cohesin and sister chromatid cohesion are not affected by deletion of *SGO1* in budding yeast mitosis (Figure S4A, B, C, [31], [32]), we hypothesized that Sgo1 might affect the centromeric localization of condensin. Indeed, we found that whereas the non-SMC condensin subunit Ycg1-GFP was enriched between SPBs in mitotic wild type cells, cells lacking Sgo1 or Rts1 failed to enrich condensin (Figure 2A, B). In contrast, condensin localization was not impaired by deletion of the second PP2A regulatory subunit Cdc55 (Figure 2A, B). Similarly, the centromeric localization of another condensin subunit, Smc2-GFP, was severely decreased in the absence of Sgo1 or Rts1 (Figure S4D, E). Condensin is also highly enriched at rDNA repeats, which can be observed as a characteristic crescent-shaped sub-nuclear signal by imaging of GFP-labelled condensin subunits [33]. This localization was not affected in any of the analyzed mutants (Figure 2A, S4D). ChIP analysis confirmed that the lack of Sgo1 did not impair the enrichment of condensin on the rDNA locus, but the centromeric and pericentric pools of FLAG-tagged Smc2 were reduced in nocodazole-arrested cells lacking Sgo1 to 30% of the wild type level (Figure 2C). The reduced levels of Smc2 at centromeric regions were also observed in *rts1*Δ cells by ChIP-qPCR experiments, although to a lesser degree in comparison to *sgo1*Δ (58% of the wild type level; Figure 2D). Condensin co-localizes on the rDNA with Lrs4 and Csm1 and its recruitment to rDNA strictly depends on these proteins [34]. Whereas we observed Csm1-GFP to localize to rDNA throughout the cell cycle as previously reported [34], no enrichment was detected between SPBs in pre-anaphase cells (Figure S5A). Our results show that condensin is localized to the pericentric region via a Sgo1/PP2A-Rts1-dependent pathway and that this localization is independent of condensin's association with rDNA.

Previously, it was shown that the lack of condensin reduces the ability to withstand the outward-directed forces of the mitotic spindle and leads to extensive centromeric stretching [16]. The finding that Sgo1 and Rts1 recruit condensin to the centromere predicts that the conformation of centromeres might be altered in *sgo1*Δ and *rts1*Δ cells. We therefore analyzed the compaction state of centromeric chromatin of chromosome 4. In wild type cells, most of the centromeres appear as a single spot that slightly separates during the process of biorientation (also called “kinetochore breathing”) and only 10% of cells contained stretched centromeres (Figure 2E, [16], [27]). In contrast, cells lacking *SGO1* or *RTS1* or carrying the *sgo1-N51I* allele contained up to 40% of stretched centromeres (Figure 2E). Since the absence of *CDC55* did not affect centromeric stretching (Figure 2E), we conclude that the centromeric localization of condensin is facilitated by Sgo1 acting in collaboration with Rts1. Thus, the lack of Sgo1 or Rts1 affects centromeric conformation similarly as the lack of condensin, further strengthening our findings that Sgo1 and to a lesser degree also PP2A-Rts1 are needed for the maintenance of functional condensin on centromeric chromatin.

Since both *sgo1*Δ and *rts1*Δ cells are impaired in their response to lack of tension on sister KTs, we asked whether condensin mutations alone show a similar phenotype. The induction of syntelic attachments by Cik1-cc overexpression impaired the growth of cells carrying the temperature-sensitive condensin alleles *smc2-8* or *ycg1-10* even at a non-restrictive temperature, thus demonstrating that these mutants fail to recognize or repair syntelic attachments (Figure 2F). To test whether the pre-anaphase centromeric functions of condensin can be separated from its anaphase functions, we used the yeast strain D1225 that expresses non-posphorylatable mutants of the condensin subunits Ycg1, Brn1, and Ycs4, which interfere specifically with condensin's function in anaphase [35]. Importantly, the presence of syntelic attachments did not affect the proliferation of the D1225 mutant strain (Figure 2F). Additionally, deletion of the Lrs4 and Csm1 proteins that localize condensin to rDNA, but not to the centromere, did not impair the ability of cells to repair syntelic attachments induced by overexpression of Cik1-cc (Figure S5B). Thus, Sgo1/PP2A-Rts1-dependent recruitment of condensin to the pericentric region is essential for the pre-anaphase chromatin conformation and for the correction of faulty MT-KT attachments. This function of condensin can be clearly separated from its anaphase-specific function in chromosome compaction as well as from its function at repetitive rDNA sequences.

### Condensin localizes to the centromere via its interaction with Sgo1 independently of PP2A-Rts1 phosphatase activity

**Figure 3:**
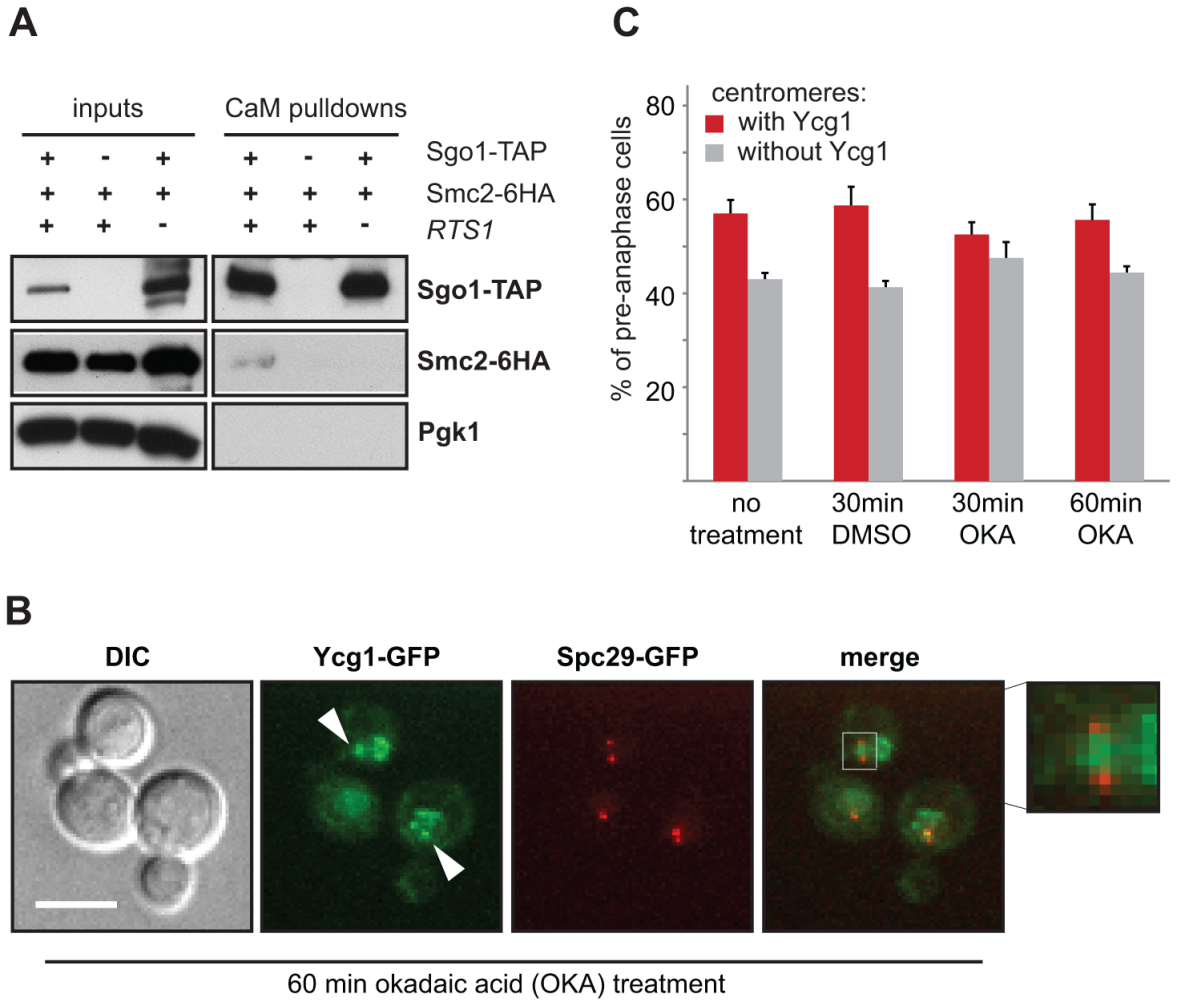
Centromeric localization of condensin does not require PP2A's phosphatase activity. (A) Sgo1-TAP pulls down the condensin subunit Smc2-6HA. Co-immunoprecipitation of Smc2-6HA is dependent on the presence of Rts1. (B) Example of Ycg1-GFP localization after 60 min OKA treatment. SPBs are visualized with Spc29-RFP. Bar – 5 µm. (C) Quantification of subcellular localization of the condensin subunit Ycg1-GFP in the presence of OKA.

To elucidate the mechanism of condensin localization to centromeres, we analyzed whether Sgo1 interacts with condensin in mitotic cells. To this end we precipitated Sgo1-TAP from yeast protein lysates. Indeed, we found that Sgo1 specifically pulls down the condensin subunit Smc2. This interaction is abolished in cells lacking Rts1, further strengthening the notion that Rts1 aids Sgo1 in condensin recruitment to the centromere (Figure 3A). The finding that Rts1 is important for the pulldown of Smc2 with Sgo1-TAP prompted us to ask whether the phosphatase activity of PP2A-Rts1 plays a role in the recruitment of condensin to centromeres. Therefore, we treated cells with okadaic acid (OKA), a potent inhibitor of the phosphatase activity of PP2A and analyzed its influence on condensin localization to centromeres in wild type cells. We found that the centromeric recruitment of the condensin subunit Ycg1-GFP is insensitive to treatment with OKA (Figure 3B, C). To exclude that this result is due to the low efficacy of the inhibitor *in vivo*, we analyzed the anaphase localization of Kin4, a well characterized target of PP2A-Rts1 [36], [37]. These experiments showed that the same concentration of OKA that did not affect Ycg1 localization efficiently delocalized Kin4 from the mother SPB within 30 min (Figure S6A, B). Similar observations were previously reported for Xenopus egg extracts and HeLa cells, where PP2A physically interacts with condensin II and recruits it to chromosomes independently of its phosphatase activity [38]. Taken together, our results suggest that Sgo1 and PP2A-Rts1 mediate condensin enrichment at the pericentric region. The recruitment of condensin does not depend on the phosphatase activity of PP2A, but rather relies on a physical interaction with Sgo1 supported by Rts1.

### Sgo1, PP2A and condensin affect correct Ipl1 localization

**Figure 4:**
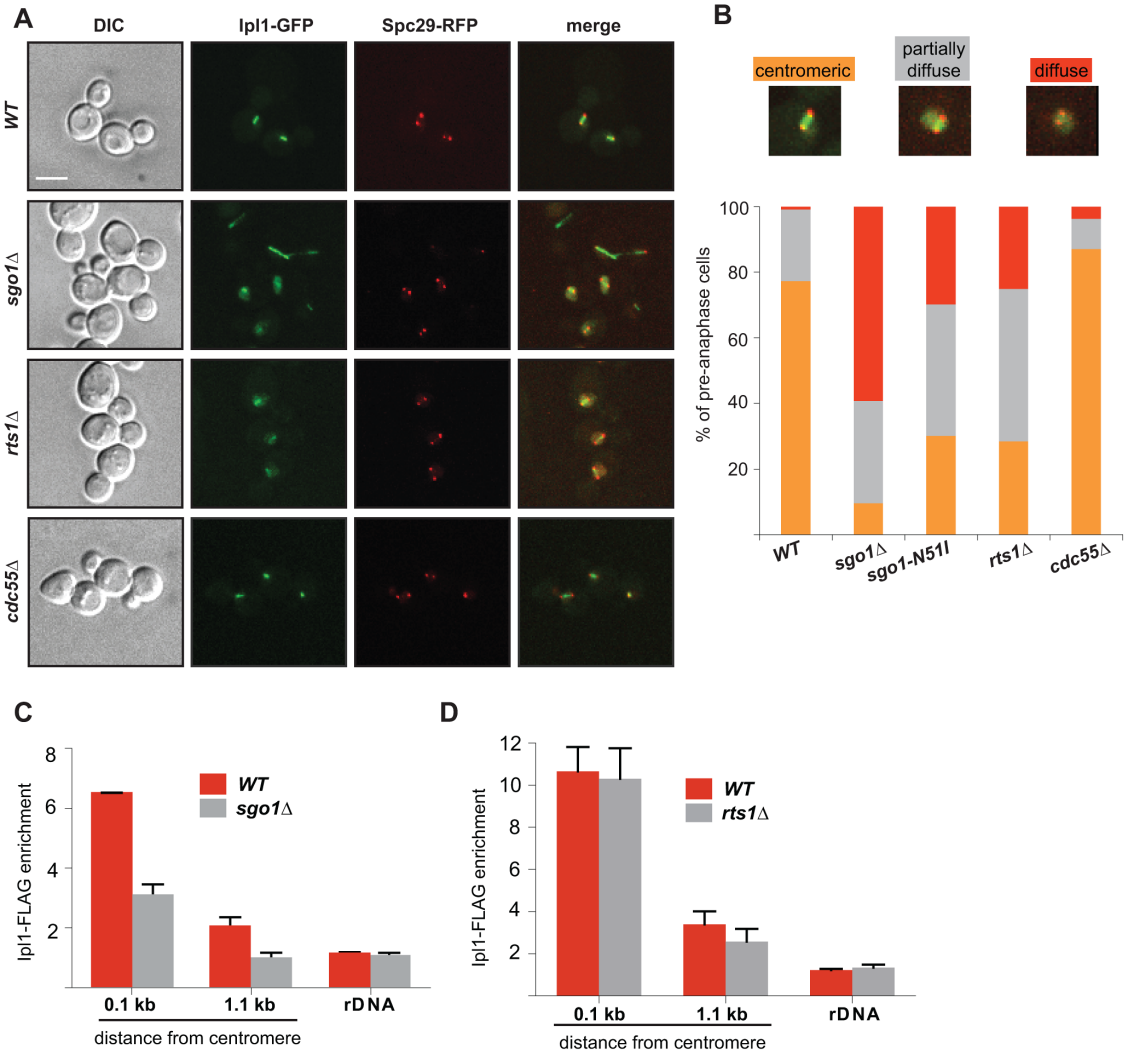
Sgo1 and Rts1 are required for the maintenance of Ipl1 localization and activity at the centromere. (A) Localization of Ipl1-GFP during mitosis in wild type cells and in cells lacking Sgo1, Rts1 or Cdc55. SPBs are visualized with Spc29-RFP. Bar – 5 µm. (B) Quantification of Ipl1-GFP localization on pre-anaphase spindles in wild type and *sgo1*Δ, *sgo1-N51I*, *rts1*Δ and *cdc55*Δ mutants (SPB distance <2 µm). Mean values of three independent experiments are shown. At least 150 cells were scored in each experiment. Top – examples of scored categories. (C) Enrichment of Ipl1-FLAG on centromeric DNA (0.1 kb away from CEN1 and 1.1 kb away from CEN4) and on rDNA (NTS1-2) normalized to the levels of Ipl1-FLAG bound to the arm of chromosome 10 in mitotic cells. ChIP-qPCR experiments of Ipl1-FLAG were performed using wild type and *sgo1*Δ cells arrested with nocodazole. Error bars represent the standard error of the mean. (D) Enrichment of Ipl1-FLAG on centromeric DNA (0.1 kb away from CEN1 and 1.1 kb away from CEN4) and on rDNA (NTS1-2) normalized to the levels of Ipl1-FLAG bound to the arm of chromosome 10 in mitotic cells. ChIP-qPCR experiments of Ipl1-FLAG were performed using wild type and *rts1*Δ cells arrested with nocodazole. Error bars represent the standard error of the mean.

Recent high-throughput analysis revealed that condensin is required for the correct localization of the conserved kinase Aurora B/Ipl1 during metaphase and anaphase [39]. Since Sgo1 and PP2A-Rts1 are essential for condensin localization, we would expect a defective Aurora B/Ipl1 localization in *sgo1*Δ and *rts1*Δ mutants as well. Visualization of Ipl1-GFP revealed that the initial recruitment of the kinase to the KTs (before separation of the SPBs) was comparable in both wild type and *sgo1*Δ mutant cells (Figure S7A). In contrast, we observed a marked difference in Ipl1 localization at pre-anaphase spindles. Ipl1 localizes exclusively to centromeres and a diffuse nuclear signal was only rarely observed in wild type pre-anaphase cells (Figure 4A, B), which is in agreement with previous observations (*e*.*g*. [40] [41]). Careful analysis revealed that whereas cells with very short pre-anaphase spindles localize Ipl1-GFP between the SPBs, the cells with a diffuse nuclear localization of Ipl1-GFP had on average longer spindles (Figure S7B). In the absence of Sgo1, 57.8% of cells exhibited a diffuse nuclear signal of Ipl1-GFP, pointing to a defect in maintaining Ipl1 at the centromere (Figure 4A, B). Moreover, the correlation between spindle length and Ipl1-GFP localization was lost in *sgo1*Δ cells (Figure S7B). Ipl1 localization was also altered in cells lacking Rts1 or carrying the *sgo1-N51I* allele, although the phenotype was milder than in the cells lacking Sgo1 (25.0% and 31.2% of cells with diffuse Ipl1 signal, respectively; Figure 4A, B). No changes were observed in cells lacking Cdc55 (Figure 4A, B). ChIP analysis in cells arrested by nocodazole treatment showed that the levels of centromeric and pericentric Ipl1-FLAG were diminished in *sgo1*Δ cells to 52% of the wild type levels (Figure 4C). In contrast, the lack of *RTS1* did not considerably impair Ipl1-FLAG recruitment to centromeres (Figure 4D). This indicates that Sgo1 might contribute to the maintenance of centromeric Ipl1 independently of Rts1 recruitment, or that, for technical reasons, the effect of PP2A-Rts1 is difficult to detect in nocodazole-arrested cells. Based on these observations we propose that Sgo1 is dispensable for the initial recruitment of Ipl1, but becomes essential for maintaining the centromeric localization of Ipl1 during the establishment of biorientation in budding yeast.

Next, we asked whether impairing the centromeric condensin pool leads to a more general defect in mitotic cells affecting the protein occupancy of the centromeric region, perhaps due to an altered DNA conformation. To test this possibility, we analysed strains carrying the temperature-sensitive condensin allele *smc2-8*. This mutation leads to a dramatic delocalization of Ycg1-GFP from centromeres at the non-permissive temperature (Figure S8A). However, we observed that Sgo1 and Rts1 localization was only marginally affected by the *smc2-8* mutation (Figure S8B, C). Markedly, almost 40% of pre-anaphase spindles showed a diffuse Ipl1-GFP localization (Figure S8D). This suggests that condensin may contribute to the maintenance of Ipl1 on centromeres.

### Both centromeric condensin and Ipl1 activity are required for the correction of syntelic attachments

**Figure 5:**
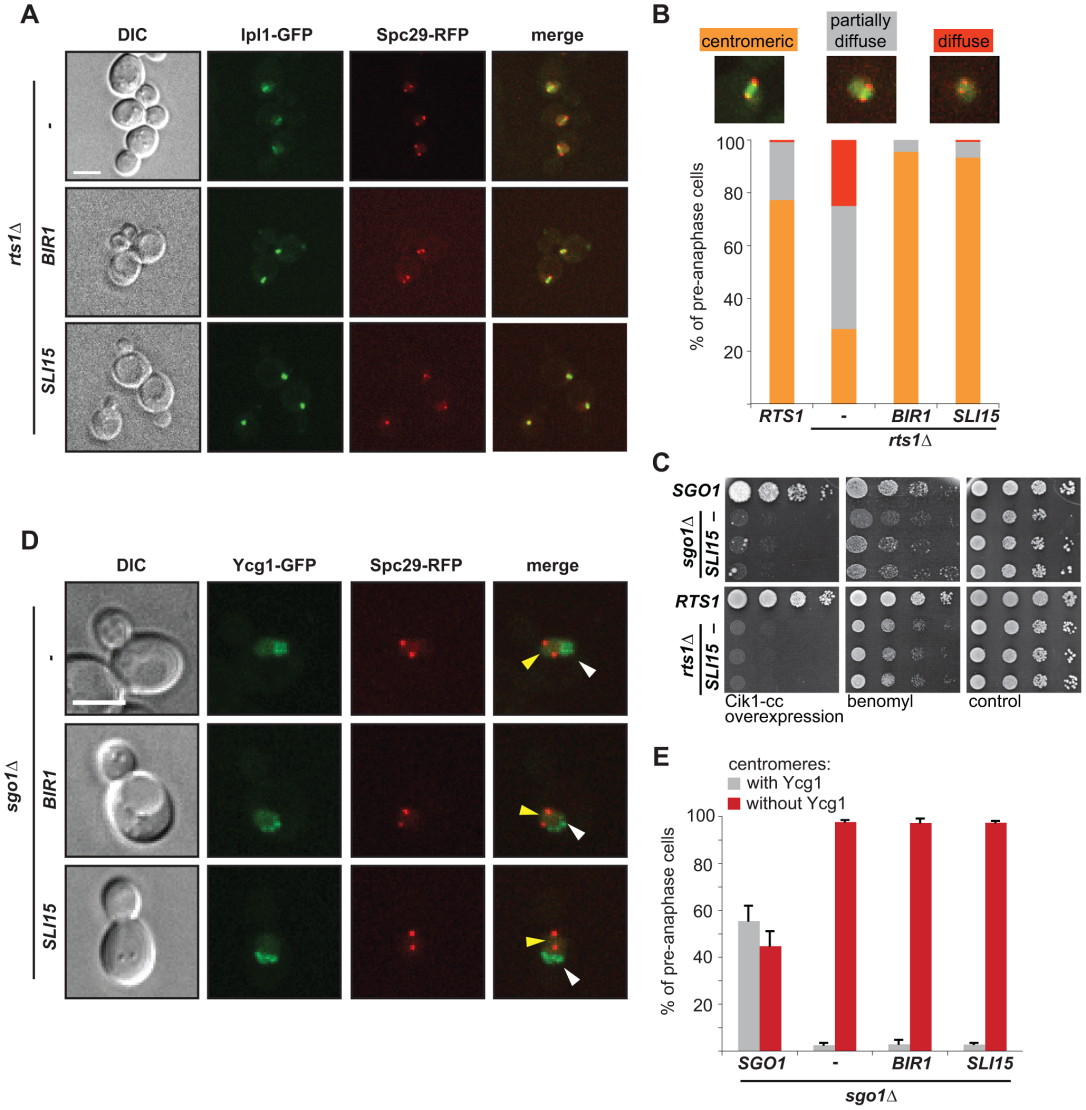
Dual role of Sgo1 in localization of condensin and Ipl1. (A) Localization of Ipl1-GFP in *rts1*Δ cells that overexpress *BIR1* or *SLI15*. SPBs are visualized with Spc29-RFP. Bar – 5 µm. (B) Quantification of Ipl1-GFP localization on pre-anaphase spindles. Means of three independent experiments are shown. At least 150 cells were scored in each experiment. (C) Growth of *sgo1*Δ and *rts1*Δ cells which overexpress *BIR1* or *SLI15* in the presence of microtubule poisons and under conditions leading to syntelic attachments. (D) Localization of the Ycg1-GFP signal in *sgo1*Δ cells which overexpress *BIR1* or *SLI15*. SPBs are visualized with Spc29-RFP. Yellow arrowheads indicate lack of centromeric localization. White arrowheads indicate Ycg1-GFP localized to rDNA. Bar – 5 µm. (E) Quantification of Ycg1-GFP localization. Only pre-anaphase spindles were scored (SPB distance <2 µm). Means with SD of three independent experiments are shown. At least 150 cells were scored in each experiment.

The data presented above suggest a linear pathway where Sgo1, PP2A and condensin cooperate to ensure the maintenance of Ipl1 activity at the centromere. At the same time, we observed one marked difference: whereas condensin localization requires both Sgo1 and to a lesser degree also Rts1 function, the localization of Ipl1 depends only on Sgo1. To further explore the functional interactions, we exploited the finding that the CPC members Bir1 and Sli15 are high-copy-number suppressors of the benomyl sensitivity and ploidy-specific lethality of *sgo1*Δ mutants [41], [42]. We asked whether the overexpression of CPC subunits would restore the reduced levels of Ipl1 on the centromere in cells lacking centromeric Rts1. Indeed, Ipl1-GFP localization between the SPBs at pre-anaphase spindles was completely rescued by overexpression of Sli15 or Bir1 (Figure 5A, B). We next tested whether the restored localization of Ipl1 eliminates the defect in correction of tensionless MT-KT attachments in *sgo1*Δ and *rts1*Δ cells. As previously observed, overexpression of Sli15 partially rescued the growth of *sgo1*Δ in the presence of microtubule-depolymerizing drugs, but we found that the growth defect of *rts1Δ* cells was not improved (Figure 5C, [41]). We suggest that this difference reflects the different roles of Sgo1 and Rts1 in the maintenance of Ipl1 on centromeric DNA. Additionally, we found that the overexpression of Sli15 cannot rescue the exquisite sensitivity of *sgo1*Δ and *rts1*Δ mutants to syntelic attachments induced by Cik1-cc overexpression (Figure 5C). Thus, although the increased abundance of Sli15 or Bir1 increases the pool of Ipl1 localized between the SPBs, this is not sufficient to repair syntelic attachments when they are generated at high frequencies.

**Figure 6:**
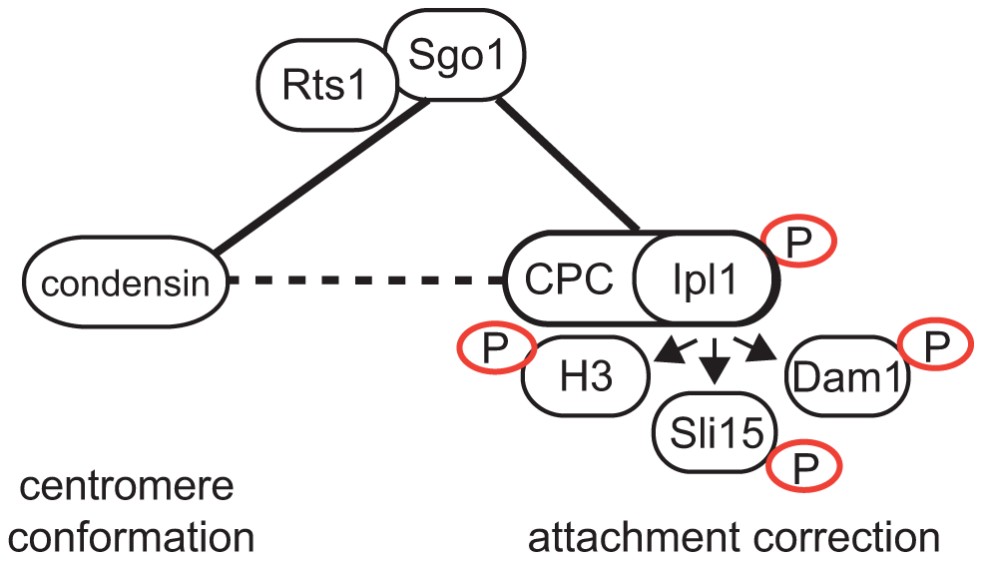
Model of Sgo1 function during establishment of biorientation. Connecting lines represent protein-protein interactions, dashed lines represent functional interactions. Full arrow depicts phosphorylation.

To determine why cells that localize Ipl1 properly, but lack centromeric Sgo1, cannot correct syntelic attachments, we analyzed the localization of the GFP-tagged condensin subunit Ycg1 in *sgo1*Δ cells overexpressing either Sli15 or Bir1. Importantly, we observed that the overexpression of CPC subunits did not restore centromeric condensin localization in *sgo1*Δ mutants (Figure 5D, E). From these results we conclude that the two functions of Sgo1 at the centromere – maintenance of the Ipl1 kinase at the MT-KT interface and providing flexibility to the pericentric region by condensin recruitment – are at least partially independent (Figure 6). Our findings illustrate that Sgo1 coordinates two essential functions at the centromere in order to facilitate chromosome biorientation during mitosis.

## Discussion

Here, we clarify the function of the Sgo1/PP2A-Rts1 interaction during the establishment of bioriented MT-KT attachments in budding yeast mitosis. We show that the Sgo1-dependent recruitment of PP2A-Rts1 is required for efficient localization of condensin to the centromere and that Sgo1 ensures the maintenance of centromeric Aurora B/Ipl1. Moreover, Sgo1 pulls down condensin in the presence of Rts1. Importantly, centromeric enrichment of both condensin and Aurora B/Ipl1 are essential for correct chromosome segregation. Our results are in agreement with the recent finding that shugoshin facilitates chromosome biorientation via condensin recruitment [43] and together suggest that Sgo1 serves as a hub protein that coordinates the molecular activities required for the biorientation of sister chromatids.

Budding yeast Sgo1, as all other members of the shugoshin protein family analyzed so far, interacts with the B56 regulatory subunit of protein phosphatase PP2A (Rts1 in budding yeast) via its N-terminal coiled-coil domain [8]–[11]. This interaction is crucial for shugoshin-mediated protection of centromeric cohesin from cleavage by separase during meiosis I and from phosphorylation-mediated removal during mitosis in vertebrates (reviewed in [44]). Yet, several lines of evidence suggest that in budding yeast, Sgo1 together with PP2A facilitates the establishment of biorientation, but by a mechanism independent of cohesin regulation. First, although lack of Sgo1 does not affect cohesion in budding yeast mitosis, the cells fail to recognize and correct improperly attached sister chromatids (Figure 1D, E, S4A, B, C, [4], [7], [41]). Moreover, cells carrying a mutation that impairs the Sgo1-Rts1 interaction fail to arrest in the presence of tensionless attachments induced by depletion of cohesin (Figure 1D, [11]). Additionally, cells lacking *RTS1* or carrying mutations that impair the Sgo1-PP2A interaction show a marked sensitivity to microtubule depolymerizing drugs (Figure 1E, S2, [11]). We also observed that lack of Rts1 leads to defects in chromosome segregation (Figure S3A, B). Very recently, it was reported that Rts1 is not required for establishment of chromosome biorientation in cells arrested in metaphase by depletion of the anaphase regulator Cdc20, although a Sgo1 mutant that has lost the ability to interact with Rts1 showed a strong phenotype [43]. This result differs from our findings that Rts1 and the Sgo1-Rts1 interaction are required for correct chromosome segregation. Thus, future experiments should focus on dissecting the functions of Sgo1 and PP2A-Rts1 during the establishment of biorientation by identification of additional separation-of-function alleles.

The correct conformation of pericentric chromatin is essential for bioriented MT-KT attachments because it provides the rigidity and at the same time elasticity necessary for chromatin to withstand the opposing forces exerted by spindle MTs [17]. The inactivation of either cohesin or condensin results in the loss of tension across KTs and in defective turnover of syntelic attachments [30], [45]. We speculated that Sgo1 together with PP2A-Rts1 might affect the conformation of pericentric chromatin during metaphase, possibly by affecting loading of condensin onto this region. An increasing body of evidence supports this idea. First, condensin, similarly to cohesin, is a member of the SMC (structural maintenance of chromosomes) protein family and is, among others, required for the structure and organization of pericentric regions [18]. Second, condensin has been implicated in tension sensing and lack of condensin abolishes the conformational changes upon tension [16], [19], [20]. Finally, the ability of the inner KT to undergo conformational changes in response to altered tension is impaired in yeast cells lacking either Sgo1, or Bub1, a mitotic kinase essential for Sgo1 localization [21]. Here we demonstrate that Sgo1 and to a lesser degree also Rts1 are required for the localization of condensin specifically to centromeric and pericentric regions, but not to rDNA (Figure 2A, B, C, D, S4D, E). Importantly, cells with defective condensin cannot proliferate in the presence of microtubule poisons or when syntelic attachments are formed at high frequencies due to Cik1-cc overexpression and this condensin function can be separated from its anaphase functions in chromosome hypercondensation (Figure 2E). We hypothesized that PP2A might inhibit the phosphorylation of condensin to prevent its premature removal, similarly as was observed for cohesin molecules [44]. However, the localization of condensin is resistant to treatment with okadaic acid, a potent inhibitor of PP2A, suggesting that phosphatase activity is not required (Figure 3B, C). This is in agreement with studies in Xenopus egg extracts and HeLa cells, where recruitment of condensin II to chromosomes relies on the presence of PP2A independently of its phosphatase activity [38]. Yet, unlike yeast condensin, the localization of condensin II in higher eukaryotes is affected by the presence of okadaic acid; only the use of a catalytically inactive but correctly localized PP2A mutant revealed that chromosomal association of PP2A, but not its phosphatase activity, is essential for the targeting of condensin II to chromatin in Xenopus [38]. The difference might be explained by the fact that centromeric PP2A-Rts1 is recruited through an interaction with Sgo1, whereas Takomoto and colleagues analyzed the recruitment of condensin II to chromatin along entire chromosomes. Future research should clarify the nature of the interaction between Sgo1 and condensin as well as the role of Rts1 in condensin recruitment.

What is the function of condensin on centromeres? One possibility is that condensin maintains the conformation of centromeric regions, thereby facilitating the intrinsic bias of budding yeast KTs to biorient on the mitotic spindle [6]. This model would predict that cells lacking centromeric condensin should create monooriented attachments more often than wild type cells. However, cells lacking Sgo1 (and hence centromeric condensin) do not show any defect in the intrinsic bias of sister kinetochores to biorient [6]. Therefore, we suggest that the inability to localize centromeric condensin impairs error sensing and correction rather than contributing to the generation of more erroneous attachements.

We considered two possible roles for condensin in the correction of tensionless attachments. First, condensin might be required to facilitate the centromeric localization of Aurora B/Ipl1. Indeed, recently it has been shown that metaphase centromeres and KTs become deformed and Aurora B is mislocalized upon depletion of either condensin I or II in vertebrate cells [20]. Similarly, budding yeast lacking condensin fail to localize Ipl1 properly to the centromere during metaphase as well as to the spindle during anaphase [39]. This finding is in agreement with our observation that the absence of Sgo1 (and hence the absence of centromeric condensin) alters the localization of Ipl1 on centromeres during the establishment of biorientation. These results might imply a linear pathway where Sgo1 recruits PP2A, which, in turn, by recruiting condensin facilitates localization of Ipl1 to the centromere. Several important observations cannot be fully reconciled with this hypothesis. First, whereas full centromeric localization of condensin requires the presence of Rts1, localization of Ipl1 likely does not (compare Figure 2 and Figure 4). Second, although the overexpression of the CPC subunits Bir1 or Sli15 partially rescues the segregation defects and benomyl sensitivity of *sgo1*Δ cells and centromeric localization of Ipl1 in *rts1*Δ cells (Figure 5A, B, C, [41]), it does not affect the localization of condensin, nor the sensitivity of *sgo1*Δ and *rts1*Δ strains to Cik1-cc overexpression (Figure 5C, D, E). Additionally, deletion of *SGO1* and mutations in condensin subunits are synthetically lethal, suggesting that they have non-overlapping functions [30]. Thus, although the functions of condensin and Ipl1 at the KT are closely linked, they are not arranged in a single linear pathway (Figure 6). We favor a model in which condensin is mainly required to establish the centromeric conformation that allows the generation of tension across bioriented KTs. Recent results suggest that condensin contributes to tension sensing by maintaining stiff, but flexible chromatin structure, which further supports our model [16].

In the wild type scenario, the majority of cells with very short spindles show an enrichment of Ipl1 between the SPBs, but with increased spindle length more cells with diffuse nuclear Ipl1 can be observed. One possible interpretation of this observation is that once tension is established, Ipl1 becomes more dynamic and is eventually released from the bioriented KTs. This is in agreement with the observation that Ipl1-GFP is often delocalized from centromeres in cells arrested in metaphase by Cdc20 depletion [40], [46]. The dynamics of Ipl1/Aurora B localization on centromeres is likely regulated by phosphorylation. A previous report demonstrated that shugoshin proteins from *S. pombe* and human cells interact with CPC subunits and this interaction depends on phosphorylation by Cdk1 [47]. Moreover, Ipl1 co-precipitates during mitosis with Sgo1 in budding yeast. Interestingly, this co-precipitation as well as centromeric Ipl1 localization was impaired in a strain carrying the mutant allele *sgo1-3A* which interferes with the Sgo1-Rts1 interaction [43]. Finally, Ipl1 is phosphorylated by the cyclin-dependent kinase Cdc28 upon anaphase onset; this phosphorylation prompts the binding of Ipl1 to Bim1, a microtubule plus-end tracking protein [46]. In the future, it should be determined whether Sgo1 affects the Cdc28-dependent phosphorylation of Aurora B/Ipl1 or other CPC subunits, thereby regulating their dynamics on the centromere during mitosis. Additionally, the role of protein phosphatases in this process remains to be characterized.

The centromeric localization and activity of Ipl1 in pre-anaphase cells is regulated by several factors. First, defective condensin impairs the localization of Ipl1 (but not vice versa [39]) and it has been shown that condensin subunits interact with Bir1, a member of the CPC [39]. Second, the overexpression of Bir1 or Sli15 localizes Ipl1 to the region between the SPBs in the absence of Rts1 (Figure 5A, B). Although we do not understand why Ipl1 localization is restored and whether under these conditions Ipl1 localizes exactly as in wild type cells, it has recently been postulated that Aurora B is tethered to the centromeric chromatin within the inner KT by Incenp/Sli15 [48]. The lack of Sgo1 also impairs the maintenance of Ipl1 localization during metaphase (Figure 4). This is in line with previous data that shugoshin proteins from human cells, fission and budding yeasts interact with the CPC, thereby contributing to the localization of Aurora B/Ipl1 at the pericentromere [43], [47]. Taken together, these results suggest the intriguing possibility that Sgo1 maintains centromeric localization of the CPC via a direct interaction (Figure 6). Lastly, the initial recruitment of Ipl1 to the centromere is independent of Sgo1 (Figure S7A, [41], [43]), indicating the existence of another recruiting mechanism, likely via the interaction of the CPC protein Bir1 with the KT protein Ndc10 [49], [50]. The fact that Aurora B/Ipl1 localization is regulated by several different mechanisms in both yeast and higher eukaryotes indicates that the CPC needs to be positioned correctly and in a timely manner in order to perform its functions. Yet, recent data suggest that even mislocalized Ipl1 can efficiently correct erroneous MT-KT attachments [51]. Further research should elucidate the exact spatio-temporal coordination of the molecular events during the establishment of biorientation. Our data show that Sgo1 is an important player during this event, serving as a hub modulating centromeric conformation and integrating phosphatase and kinase activities at the metaphase spindle.

## Materials and Methods

### Yeast strains and growth

All yeast strains used in this study are listed in Supporting Table S1 and derived from the genetic background of W303 (*leu2-3, 112 trp1-1 can1-100 ura3-1 ade2-1 his3-11,15*) or BY4741 (*his3*Δ*1 leu2*Δ*0 met15*Δ*0 ura3*Δ*0*). Gene deletions and epitope-tagging were performed using standard PCR-based methods. Cells were grown in either full (YP; 1% yeast extract, 2% Bacto-Peptone) or synthetic complete (SC; 1.34% yeast nitrogen base, 0.04% complete synthetic mix) medium supplemented with 2% glucose (YPD), 2% raffinose (YPR) or 2% galactose (YPG). Cells were arrested in G1 using 10 µM α-factor (Core Facility, Max Planck Institute of Biochemistry, Martinsried, Germany) or in mitosis using 30 µg/ml nocodazole (Santa Cruz Biotechnology). Mutants were grown at 25 °C to reduce chromosome missegregation. For viability assays cells were grown overnight in full medium, diluted to an OD_600_ of 0.3 and tenfold serial dilutions were spotted on YPD/YPG plates or on SC plates containing indicated concentrations of benomyl and nocodazole, respectively. The phosphatase inhibitor okadaic acid was used at concentrations of 10 or 20 µM as stated for the individual experiments.

### Plasmid construction

All plasmids used in this study are listed in Supporting Table S2. Construction of plasmids was performed as described previously using standard cloning procedures. A fragment encoding amino acids 81–360 of Cik1 was cloned into the multiple cloning site (MCS) of pRS406 containing the inducible *GAL1* promoter with a C-terminal TAP- or GFP-tag. *SGO1* was cloned with its endogenous promoter into pRS405 with a C-terminal TAP-tag. Plasmids encoding the Mtw1-fusion proteins were constructed by cloning *MTW1* with its endogenous promoter adjacent to the corresponding gene into the MCS of pRS405. Chromosomal integration of plasmids encoding Sgo1- or Mtw1-fusion proteins was targeted into the endogenous locus using restriction enzymes cutting in the respective promoter region. Point mutations were introduced using the PCR-based Quick Change (Stratagene) site-directed mutagenesis approach.

### Protein techniques

Protein extracts of *S. cerevisiae* were prepared either by glass bead lysis or alkaline lysis followed by TCA precipitation. Proteins were separated by SDS-PAGE, transferred to PVDF or nitrocellulose membranes and detected using antibodies according to standard protocols. Commercially available antibodies were used to detect individual proteins: myc (9E10, Santa Cruz Biotechnology), HA (Y-11, Santa Cruz Biotechnology), PAP (Sigma-Aldrich), Clb2 (y-180, Santa Cruz Biotechnology), Pgk1 (Invitrogen).

### *In vivo* interaction analysis

Protein extracts from *S. cerevisiae* were prepared by glass bead lysis in a lysis buffer (10 mM Hepes, 200 mM KCl, 1 mM MgCl2, 1 mM DTT, 1 mM PMSF, 0.5% Triton X-100, supplemented with Roche Protease Inhibitor Mix) followed by centrifugation (100000 g, 45 min, 4°C). Sgo1-TAP was purified by incubation of approx. 3.5 mg protein extract with pre-washed calmodulin-coated beads (GE Healthcare). Beads were washed using lysis buffer containing increasing salt concentrations (up to 600 mM KCl) and finally eluted in the presence of 5 mM EGTA. Proteins were precipitated with TCA and co-purified proteins were analyzed by SDS-PAGE followed by immunoblotting.

### PP2A – His-Sgo1-pulldown experiments

Heterotrimeric PP2A complexes were purified from mitotic lysates of *S. cerevisiae* via Rts1- or Cdc55-TAP fusion proteins using calmodulin-coupled agarose as described above. Equal amounts of PP2A complexes were incubated with recombinant Ni-NTA agarose-bound His-Sgo1 fragments containing the conserved coiled-coil domain (amino acids 1–340) in a pulldown buffer (50 mM Tris, 300 mM NaCl, 10 mM imidazole, 1 mM PMSF supplemented with Roche protease inhibitor mix) for 2 hours. Proteins were eluted by boiling in SDS sample buffer and subjected to SDS-PAGE and immunoblotting to detect the bound PP2A subunits.

### Fluorescence microscopy

Images of cells were obtained using a fully automated Zeiss inverted microscope (AxioObserver Z1) equipped with a MS-2000 stage (Applied Scientific Instrumentation, USA), a CSU-X1 spinning disk confocal head (Yokogawa, Herrsching), LaserStack Launch with selectable laser lines (Intelligent Imaging Innovations, USA) and an X-CITE Fluorescent Illumination System. Images were captured using a CoolSnap HQ camera (Roper Scientific, Canada) under the control of the Slidebook software (Intelligent Imaging Innovations, USA). All fluorescence signals were imaged with a 63x oil objective. A total of 10 z-stacks were collected and each optical section was 0.4 µm thick. Projected images were used for display. Only cells with spindles shorter than 2 µm and fully localized in the mother cell were scored to evaluate the localization in pre-anaphase. For Ipl1-GFP microscopy we defined three types of Ipl1-GFP localization: “centromeric” – fluorescence signal only in the area between the SPBs, “partially diffuse” – diffuse nuclear fluorescence signal and increased fluorescence intensity between the SPBs and “diffuse” – only diffuse nuclear fluorescence signal.

### Chromatin-immunoprecipitation (ChIP)

Chromatin immunoprecipitation (ChIP) was performed as previously described [52]. In brief, 100 ml of exponentially growing yeast cultures were arrested with 20 µg/ml nocodazole for 3 h at room temperature and subsequently cross-linked with formaldehyde at a final concentration of 1%. The cross-linking reaction was stopped with glycine; the cells were harvested and lysed using Silica beads. Chromatin was sheared to 300–500 bp fragments by water-bath sonification (Bioruptor UCD-200, Diagenode). FLAG-tagged proteins were immunoprecipitated using the monoclonal ANTI-FLAG antibody coupled to superparamagnetic beads (Sigma-Aldrich, M8823). DNA was recovered by phenol/chloroform extraction followed by ethanol precipitation. Quantitative RT-PCR was performed using the Light Cycler LC480 system (Roche) to evaluate the enrichment of analyzed proteins. The ratio of DNA-IP to DNA-Input was calculated for centromeric/pericentromeric regions (0.1 kb away from CEN1, 1.1 kb away from CEN4 and 5 kb away from CEN12) as well as for the rDNA locus (NTS1-2). The relative enrichment was calculated by normalization to the IP/Input ratio for a control locus on the arm of chromosome 10. Three independent immunoprecipitation experiments from metaphase-arrested cells were performed for FLAG-tagged Smc2 and Ipl1; Rts1 and Mcd1 ChIP experiments were performed twice.

## Supporting Information

Figure S1 **Functional interactions between Sgo1 and PP2A**. (A) The heterotrimeric PP2A-Rts1 complex was purified from mitotic protein lysates via Rts1-TAP. To analyze the interaction between Rts1 and Sgo1, the purified PP2A-Rts1 complex was loaded either on the Ni-NTA-bound wild type or mutant His-N-terminal fragment of Sgo1 (+) or on empty Ni-NTA beads (-) as a negative control. (B) The heterotrimeric PP2A-Cdc55 complex was purified from mitotic protein lysates via Cdc55-TAP (note that Tpd3-9myc could not be successfully visualized in this pulldown since it co-migrates with Cdc55-TAP). To analyze the interaction between Cdc55 and Sgo1, the purified PP2A-Cdc55 complex was loaded either on the Ni-NTA-bound wild type or mutant His-N-terminal fragment of Sgo1 (+) or on empty Ni-NTA beads (-) as a negative control. (C) Enrichment of Rts1-FLAG on centromeric DNA (0.1 kb away from CEN1, 1.1 kb away from CEN4 and 5.0 kb away from CEN12) and on rDNA (NTS1-2) normalized to the levels of Rts1-FLAG bound to the arm of chromosome 10 in mitotic cells. ChIP-qPCR experiments of Rts1-FLAG were performed using wild type and *sgo1*Δ cells arrested with nocodazole. Error bars represent the standard error of the mean. (D) Genetic interactions between *SGO1, CDC55, RTS1* and *sgo1-N51I* mutants.
(EPS)

Figure S2 **Genetic interactions between Sgo1 and PP2A**. (A) Sensitivity of *SGO1*-deficient cells and mutants to the microtubule depolymerizing drug benomyl. (B) Schematic depiction of Sgo1 and its domains. *sgo1-N51I* impairs interaction with PP2A-Rts1, *sgo1-T379D* mutation impairs localization to the centromere. (C) Sensitivity of strains with Mtw1-fusion proteins to the microtubule depolymerizing drug benomyl. (D) Schematic representation of the fusion proteins used in Figure 1C and Supporting Figures 1C, E. (E) Sensitivity of strains expressing the Sgo1-N51I-Cdc55-fusion protein to the microtubule depolymerizing drug benomyl. (F) Sensitivity of a strain overexpressing Cdc55 or Rts1 from the galactose inducible *GAL1* promoter to the microtubule depolymerizing drug benomyl. (G) Expression levels of the overexpressed p*GAL1*-Rts1-TAP.
(EPS)

Figure S3 **Recruitment of Rts1 to the kinetochores via Sgo1 is required for correction of erroneous (tensionless) attachments**. (A) Chromosome segregation (centromeres of chromosome 4 labeled by tetO/TetR-GFP) in cells released from nocodazole-induced arrest. Cells were collected 75 min after the release from nocodazole arrest into fresh medium containing α-factor (to prevent entry into another cell cycle), fixed with 3.7% formaldehyde and imaged. A mean of three experiments with standard deviation is shown. Top: Schematic depiction of the experiment. (B) Time course of the establishment of biorientation after the release from nocodazole-induced arrest. Cells were released from cell cycle arrest into fresh medium containing α-factor (to prevent entry into another cell cycle) and samples were collected at indicated time points, fixed with 3.7% formaldehyde and imaged. A mean of four experiments with standard deviation is shown. Red line – rate of SPB separation; green line – rate of chromosome biorientation. Micrographs: left: examples of cells with and without bioriented chromosomes; right: examples of cells correctly segregating and missegregating chromosome 4. Bar – 1 µm. (C) Progression through the cell cycle upon induction of the spindle assembly checkpoint by nocodazole. Top – Schematic depiction of the experimental setup. Cells were synchronized in G1 by α-factor and released into medium containing nocodazole. Samples were collected at indicated time points.
(EPS)

Figure S4 **Localization of condensin and cohesin subunits in *sgo1*Δ and *rts1*Δ cells**. (A) Localization of the cohesin subunit Smc3-GFP in pre-anaphase wild type, *sgo1*Δ and *rts1*Δ cells. SPBs are marked with Spc29-RFP. Bar – 5 µm. (B) Quantification of the localization of the cohesin subunit Smc3-GFP in wild type, *sgo1*Δ and *rts1*Δ cells. Means with SD of three independent experiments are shown. At least 150 cells were scored in each experiment. (C) Enrichment of Mcd1-FLAG on centromeric DNA (0.1 kb away from CEN1, 1.1 kb away from CEN4 and 5.0 kb away from CEN12) and on rDNA (NTS1-2) normalized to the levels of Mcd1-FLAG bound to the arm of chromosome 10 in mitotic cells. ChIP-qPCR experiments of Mcd1-FLAG were performed using wild type and *sgo1*Δ cells arrested with nocodazole. Error bars represent the standard error of the mean. (D) Localization of the condensin subunit Smc2-GFP in wild type, *sgo1*Δ and *rts1*Δ cells. SPBs are marked with Spc29-RFP. Yellow arrowhead - centromeric Smc2-GFP. Bar – 5 µm. (E) Quantification of the localization of the condensin subunit Smc2-GFP in wild type and *sgo1*Δ cells. Means with SD of three independent experiments are shown. At least 150 cells were scored in each experiment.
(EPS)

Figure S5 **The Lrs4-Csm1 complex is not involved in the establishment of biorientation**. (A) Localization of Csm1-GFP. Yellow arrowheads indicate the absence of Csm1-GFP from the centromere/kinetochore. White arrowheads indicate rDNA localization of Csm1-GFP. Bar – 5 µm. (B) Deletion of the *CSM1* and *LRS4* genes does not lead to a growth defect in the presence of high levels of syntelic attachments.
(EPS)

Figure S6 **Localization of Kin4-GFP upon treatment with a PP2A inhibitor**. (A) Localization of Kin4-GFP in cells with and without treatment with okadaic acid. White arrow indicates the correct localization of Kin4-GFP to the mother spindle pole during anaphase. Bar – 5 µm. (B) Quantification of the changes in the Kin4 localization upon treatment with okadaic acid (20 µM). Mean of three independent experiments with standard deviation is shown.
(EPS)

Figure S7 **Analysis of Ipl1 localization in cells lacking *SGO1*.**(A) Localization of Ipl1-GFP in small budded cells in wild type and in cells lacking Sgo1. Bar – 5 µm. (B) Dependency of Ipl1-GFP localization on the length of the mitotic spindle (measured as distance between SPBs marked with Spc29-RFP). Only mitotic spindles shorter than 2 µm were counted. Means of two independent experiments are shown. At least 100 cells were counted in each experiment.
(EPS)

Figure S8 **Localization of Sgo1-GFP, Rts1-GFP, Ipl1-GFP and Ycg1-GFP in *smc2-8* cells**. (A) Control analysis of Ycg1-GFP at the pre-anaphase spindles confirms the condensin deficiency in *smc2-8* mutants at high temperature. (B) Localization of Sgo1-GFP at the pre-anaphase spindles is not markedly altered in cells with defective condensin. (C) Localization of Rts1-GFP at the pre-anaphase spindles is only slightly altered in cells with defective condensin, but the effect is not temperature-dependent. (D) Localization of Ipl1-GFP at the pre-anaphase spindles is impaired in cells with defective condensin. Means of three independent experiments (two in Ipl1-GFP) and standard deviation (not in Ipl1-GFP) are shown.
(EPS)

Table S1 **Yeast strains used in this study.**
(DOC)

Table S2 **Plasmids used in this study.**
(DOC)
