# Supplementary material for: Sgo1 Regulates Both Condensin and Ipl1/Aurora B to Promote Chromosome Biorientation
Source: PLoS Genet. 2014 Jun 19;10(6):e1004411. doi: 10.1371/journal.pgen.1004411 (PMC4063673; doi:10.1371/journal.pgen.1004411)
Supplement: Table S1 — Yeast strains used in this study. (DOC) [file pgen.1004411.s009.doc]

| Strain | Background | Relevant genotype | Source | Figure |
| --- | --- | --- | --- | --- |
| 67 | W303 | *MATa ade2-1 can1-100 his3-11 leu2-3,112 trp1-1 ura3-1* |  | S3D |
| 69 | W303 | *MATa sgo1∆*::*hphNT1* | This study | 1C, S3D |
| 116 | W303 | *MATa sgo1*::*hphNT1* *SGO1*-TAP-*LEU2* | This study | 1C |
| 152 | W303 | *MATa sgo1*::*hphNT1* *SGO1*-eGFP-*HIS3MX6* *SPC29*-RFP-*kanMX* | This study | 1A |
| YZ1221 | W303 | *MATa sgo1*::*hphNT1* *sgo1*-N51I-GFP-LEU2 *SPC29*-RFP-*kanMX* | This study | 1A |
| 343 | W303 | *MATa sgo1*::hphNT1 *RTS1*-eGFP-*HIS3MX6* *SGO1*-TAP-*LEU2* *SPC29*-RFP-*natNT2* | This study | 1B |
| 344 | W303 | *MATa sgo1*::*hphNT1* *RTS1*-eGFP-*HIS3MX6* *sgo1*-N51I-TAP-*LEU2* *SPC29*-RFP-*natNT2* | This study | 1B |
| 295 | W303 | *MATa sgo1*::*hphNT1* *MTW1*-*RTS1*-*LEU2* | This study | 1C |
| 294 | W303 | *MATa sgo1*::*hphNT1* *MTW1*-*CDC55-LEU2* | This study | 1C |
| YZ1359 | W303 | *MATa sgo1*::*hphNT1* *SGO1*-TAP-*LEU2* *pGAL1*-*cik1*-cc-TAP-*URA3* | This study | 1E, 2F |
| YZ1360 | W303 | *MATa* *sgo1::hphNT1 pGAL1*-*cik1*-cc-TAP-*URA3* | This study | 1E, 2F |
| YZ1361 | W303 | *MATa rts1*::*kanMX4 pGAL1*-*cik1*-cc-TAP-*URA3* | This study | 1E, 2F |
| YZ1364 | W303 | *MATa sgo1*::*hphNT1* *sgo1*-N51I-TAP-*LEU2* *pGAL1*-*cik1*-cc-TAP-*URA3* | This study | 1E |
| 189 | BY4741 | *MATa sgo1*::*hphNT1* *pMCD1*::*natNT2*-*pGAL1*-3HA *SGO1*-TAP-*LEU2 PDS1*-9myc-*HIS3MX6* | This study | 1D, S3C |
| 191 | BY4741 | *MATa sgo1*::*hphNT1* *pMCD1*::*natNT2*-*pGAL1*-3HA *PDS1*-9myc-*HIS3MX6* | This study | 1D, S3C |
| 195 | BY4741 | *MATa sgo1*::*hphNT1* *pMCD1*::*natNT2*-*pGAL1*-3HA *PDS1*-9myc-*HIS3MX6 sgo1*-N51I-TAP-*LEU2* | This study | 1D, S3C |
| 366 | BY4741 | *pMCD1*::*natNT2*-*pGAL1*-3HA *PDS1*-9myc-*HIS3MX6* *rts1*::*kanMX4* | This study | 1D |
| 284 | W303 | *MATa sgo1*::*hphNT1* *pPPH22*::*natNT2*-*pADH1*-3HA *TPD3*-9myc-*HIS3MX6* *RTS1*-TAP-*TRP1* | This study | S1A |
| 283 | W303 | *MATa sgo1*::*hphNT1* *pPPH22*::*natNT2*-*pADH1*-3HA *TPD3*-9myc-*HIS3MX6* *CDC55*-TAP-*TRP1* | This study | S1B |
| YZ1471 | W303 | *MATa RTS1*-3xFLAG-*natNT2* | This study | S1C |
| YZ1472 | W303 | *MATa sgo1*::*hphNT1 RTS1*-3xFLAG-*natNT2* | This study | S1C |
| YZ1298 | W303 | *MATa rts1*::*kanMX4* | This study | S1D |
| 314 | W303 | *MATa sgo1*::*hphNT1 rts1*::*TRP1* | This study | S1D |
| 370 | W303 | *MATa sgo1*::*hphNT1 rts1*::*TRP1 sgo1*-N51I-TAP-*LEU2* | This study | S1D |
| 313 | W303 | *MATa sgo1::hphNT1 SGO1-wt-TAP MATa cdc55::HIS3MX6* | This study | S1D |
| 298 | W303 | *MATa sgo1::hphNT1 cdc55::HIS3MX6* | This study | S1D |
| 473 | W303 | *MATa sgo1 hphNT1 cdc55:: sgo1*-N51I-TAP-*LEU2* | This study | S1D |
| 174 | W303 | *MATa sgo1*::*hphNT1 sgo1*-N51I-TAP-*LEU2* | This study | S2A |
| 118 | W303 | *MATa sgo1*::*hphNT1 sgo1*-T379D-TAP-*LEU2* | This study | S2A |
| 252 | W303 | *MATa sgo1*::*hphNT1* *MTW1*-*SGO1*-eGFP-*LEU2* | This study | S2C |
| 257 | W303 | *MATa sgo1*::*hphNT1* *MTW1*-eGFP-*LEU2* | This study | S2C |
| 262 | W303 | *MATa sgo1*::*hphNT1 sgo1*-N51I-*CDC55*-*LEU2* | This study | S2E |
| YZ1244 | W303 | *MATa sgo1*::*hphNT1 SGO1*-TAP-*LEU2* [*CEN*, *URA3*, *pGAL1*-*CDC55*-TAP] | This study | S2F |
| YZ1243 | W303 | *MATa sgo1*::*hphNT1* [*CEN*, *URA3*, *pGAL1*-*CDC55*-TAP] | This study | S2F |
| YZ1295 | W303 | *MATa sgo1*::*hphNT1 SGO1*-TAP-*LEU2 pGAL1*-*RTS1*-TAP-*URA3* | This study | S2F |
| YZ1296 | W303 | *MATa sgo1*::*hphNT1 pGAL1*-*RTS1*-TAP-*URA3* | This study | S2F |
| 325 | W303 | *MATa rts1*::*kanMX4 pRTS1*-*RTS1*-*URA3* | This study | S2G |
| YZ1299 | W303 | *MATa pGAL1*-*RTS1*-TAP-*URA3* | This study | S2G |
| 383 | W303 | *MATa sgo1*::*hphNT1 SGO1*-TAP-*LEU2* *YCG1*-eGFP-*HIS3MX6* *SPC29*-RFP-*natNT2* | This study | 2A, 2B, 3B, 3C |
| 384 | W303 | *MATa sgo1*::*hphNT1* *YCG1*-eGFP-*HIS3MX6* *SPC29*-RFP-*natNT2* | This study | 2A, 2B |
| 389 | W303 | *MATa sgo1*::*hphNT1* *sgo1*-N51I-TAP-*LEU2* *YCG1*-eGFP-*HIS3MX6* *SPC29*-RFP-*natNT2* | This study | 2A, 2B |
| 411 | W303 | *MATa sgo1*::*hphNT1* *SGO1*-wt-TAP-*LEU2* *rts1*::*kanMX4* *YCG1*-eGFP-*HIS3MX6* *SPC29*-RFP-*natNT2* | This study | 2A, 2B |
| 464 | W303 | *MATa sgo1*::*hphNT1* *SGO1*-wt-TAP-*LEU2* *cdc55*::HIS3MX6 *YCG1*-eGFP-*TRP1* *SPC29*-RFP-*natNT2* | This study | 2A, 2B |
| YZ1442 | W303 | *MATa SMC2*-3xFLAG-*natNT2* | This study | 2C, 2D |
| YZ1443 | W303 | *MATa sgo1*::*hphNT1 SMC2*-3xFLAG-*natNT2* | This study | 2C |
| YZ1488 | W303 | *MATa rts1*::*kanMX4 SMC2*-3xFLAG-*natNT2* | This study | 2D |
| YZ1222 | BY4741 | *MATa his3*::TetR-GFP-*HIS3 CENIV*::TetO-*URA3 sgo1*::*hphNT1 SGO1*-TAP-*LEU2* *SPC29*-RFP-*kanM4X* | This study | 2E, S3A, S3B |
| YZ1226 | BY4741 | *MATa his3*::TetR-GFP-*HIS3 CENIV*::TetO-*URA3 sgo1*::*hphNT1* *SPC29*-RFP-*kanMX4* | This study | 2E, S3A, S3B |
| YZ1227 | BY4741 | *MATa his3*::TetR-GFP-*HIS3 CENIV*::TetO-*URA3 sgo1*::*hphNT1 sgo1*-N51I-TAP-LEU2 *SPC29*-RFP-*kanMX* | This study | 2E, S3A, S3B |
| 386 | BY4741 | *MATa his3*::TetR-GFP-*HIS3 CENIV*::TetO-*URA3 rts1*::*natNT2* *SPC29*-RFP-*kanMX* | This study | 2E, S3A, S3B |
| 385 | BY4741 | *MATa his3*::TetR-GFP-*HIS3 CENIV*::TetO-*URA3 rts1*::*LEU2* *SPC29*-RFP-*kanMX* | This study | S3A, S3B |
| 387 | BY4741 | *MATa his3*::TetR-GFP-*HIS3 CENIV*::TetO-*URA3 rts1*::*hphNT1* *SPC29*-RFP-*kanMX* | This study | S3A, S3B |
| 489 | BY4741 | *MATa his3*::TetR-GFP-*HIS3 CENIV*::TetO-*URA3 rts1*::*natNT2* *SPC29*-RFP-*kanMX* [2µ, *LEU2*, *RTS1*] | This study | S3B |
| 467 | BY4741 | *MATa his3*::TetR-GFP-*LEU2 CENIV*::TetO-*URA3 cdc55*::HIS3MX6 *SPC29*-RFP-*kanMX4* | This study | 2E |
| YZ1414 | W303 | *brn1*-*539*-3HA *ycg1*-*488*-3HA *ycs4*-*543*-13MYC *cdc15-2 pGAL1*-*cik1*-cc-eGFP-*HIS3* | This study | 2F |
| YZ1419 | W303 | *MATα ycg1-10* *pGAL1*-*cik1*-cc-TAP-*URA3* | This study | 2F |
| YZ1418 | W303 | *MATa smc2-8* *pGAL1*-*cik1*-cc-TAP-*URA3* | This study | 2F |
| 483 | W303 | *MATa sgo1*::*hphNT1 SGO1*-TAP-*LEU2 SMC2*-6HA-*HIS3MX*6 *RTS1*-9myc-*TRP1* | This study | 3A |
| 486 | W303 | *MATa SMC2*-6HA-*HIS3MX*6 *RTS1*-9myc-*TRP1* | This study | 3A |
| 488 | W303 | *MATa sgo1*::*hphNT1 SGO1*-TAP-*LEU2 SMC2*-6HA-*HIS3MX*6 *rts1*::*natNT2* | This study | 3A |
| 400 | BY4741 | *MATa SMC3*-eGFP-*HIS3MX6 SPC29*-RFP-*natNT2* | This study | S4A, S4B |
| 409 | BY4741 | *MATa SMC3*-eGFP-*HIS3MX6 SPC29*-RFP-*natNT2 sgo1*::*hphNT1* | This study | S4A, S4B |
| 410 | BY4741 | *MATa SMC3*-eGFP-*HIS3MX6 SPC29*-RFP-*natNT2 rts1*::*hphNT1* | This study | S4A, S4B |
| YZ1473 | W303 | *MATa MCD1*-3xFLAG-*natNT2* | This study | S4C |
| YZ1474 | W303 | *MATa sgo1*::*hphNT1 MCD1*-3xFLAG-*natNT2* | This study | S4C |
| 442 | W303 | *MATa SPC29*-RFP-*natNT2* *SMC2*-eGFP-*HIS3MX6* | This study | S4D, S4E |
| 443 | W303 | *MATa sgo1*::*hphNT1 SPC29*-RFP-*kanMX SMC2*-eGFP-*HIS3MX6* | This study | S4D, S4E |
| 472 | W303 | *MATa rts1*::kanMX4 *SPC29*-RFP-*natNT2 SMC2*-eGFP-*HIS3MX6* | This study | S4D, S4E |
| 380 | W303 | *MATa sgo1*::*hphNT1* *SGO1*-TAP-*LEU2* *IPL1*-eGFP-*HIS3MX6* *SPC29*-RFP-*natNT2* | This study | 4A, 4B, 4D, S7A, S7B |
| 381 | W303 | *MATa sgo1*::*hphNT1* *IPL1*-eGFP-*HIS3MX6* *SPC29*-RFP-*natNT2* | This study | 4A, 4B, S7A, S7B |
| 382 | W303 | *MATa rts1*::*kanMX4* *IPL1*-eGFP-*HIS3MX6* *SPC29*-RFP-*natNT2* | This study | 4A, 4B, 5B |
| 390 | W303 | *MATa sgo1*::*hphNT1* *sgo1*-N51I-TAP-LEU2 *IPL1*-eGFP-*HIS3MX6* *SPC29*-RFP-*natNT2* | This study | 4B |
| 463 | W303 | *MATa cdc55*::HIS3MX6 *IPL1*-eGFP-*TRP1* *SPC29*-RFP-*natNT2* | This study | 4A, 4B |
| YZ1444 | W303 | *MATa IPL1*-3xFLAG-*natNT2* | This study | 4C, 4D |
| YZ1453 | W303 | *MATa sgo1*::*hphNT1 IPL1*-3xFLAG-*natNT2* | This study | 4C |
| YZ1489 | W303 | *MATa rts1*::*kanMX4 IPL1*-3xFLAG-*natNT2* | This study | 4D |
| 434 | W303 | *MATa CSM1*-eGFP-*HIS3MX6 SPC29*-RFP-*natNT2* | This study | S5A |
| YZ1423 | BY4741 | *csm1*::*kanMX4* *pGAL1*-*cik1*-*cc*-TAP-*URA3* | This study | S5B |
| YZ1424 | BY4741 | *lrs4*::*kanMX4* *ura3*::pRS406-*URA3*-*pGAL1*-*cik1*-*cc*-TAP | This study | S5B |
| 388 | W303 | *MATa rts1*::*kanMX4* *RTS1*-TAP-*URA3* *IPL1*-eGFP-*HIS3MX6* *SPC29*-RFP-*natNT2* | This study | 5B |
| 416 | W303 | *MATa rts1*::*kanMX4* *IPL1*-eGFP-*HIS3MX6* *SPC29*-RFP-*natNT2* [2µ, *URA3*, *BIR1*] | This study | 5A, 5B |
| 417 | W303 | *MATa rts1*::*kanMX4* *IPL1*-eGFP-*HIS3MX6* *SPC29*-RFP-*natNT2* [2µ, *LEU2*, *SLI15*] | This study | 5A, 5B |
| YZ1425 | W303 | *MATa sgo1*::*hphNT1 pGAL1*-*cik1*-cc-TAP-*URA3* [2µ, *LEU2*, *SLI15*] | This study | 5C |
| YZ1476 | W303 | *MATa sgo1*::*hphNT1 pGAL1*-*cik1*-cc-TAP-*URA3* [2µ, *LEU2*] | This study | 5C |
| YZ1475 | W303 | *MATa rts1*::*kanMX4 pGAL1*-*cik1*-cc-TAP-*URA3* [2µ, *LEU2*, *SLI15*] | This study | 5C |
| YZ1477 | W303 | *MATa rts1*::*kanMX4 pGAL1*-*cik1*-cc-TAP-*URA3* [2µ, *LEU2*] | This study | 5C |
| 384 | W303 | *MATa sgo1*::*hphNT1* *SGO1*-TAP-*LEU2* *YCG1*-eGFP-*HIS3MX6* *SPC29*-RFP-*natNT2* | This study | 5E |
| 383 | W303 | *MATa sgo1*::*hphNT1* *YCG1*-eGFP-*HIS3MX6* *SPC29*-RFP-*natNT2* | This study | 5D, 5E |
| 413 | W303 | *MATa sgo1*::*hphNT1* *YCG1*-eGFP-*HIS3MX6* *SPC29*-RFP-*natNT2* [2µ, *URA3*, *BIR1*] | This study | 5D, 5E |
| 412 | W303 | *MATa sgo1::hphNT1 YCG1-*eGFP*-HIS3MX6 SPC29-*RFP*-natNT2* [2µ, *LEU2*, *SLI15*] | This study | 5D, 5E |
| 474 | BY4741 | *MATa KIN4*-eGFP-*HISMX6 SPC29*-RFP-natNT2 | This study | S6A, S6B |
| 468 | W303 | *MATa smc2-8 YCG1-eGFP-His3MX6 SPC29-RFP-natNT2* | This study | S8A |
| 469 | W303 | *MATa smc2-8 IPL1-eGFP- His3MX6 SPC29-RFP- natNT2* | This study | S8B |
| 470 | W303 | *MATa smc2-8 SGO1-eGFP- His3MX6 SPC29-RFP- natNT2* | This study | S8C |
| 444 | W303 | *MATa smc2-8 RTS1-eGFP-TRP1 SPC29-RFP- natNT2* | This study | S8D |
|  |  |  |  |  |
