# Supplementary material for: Sgo1 Regulates Both Condensin and Ipl1/Aurora B to Promote Chromosome Biorientation
Source: PLoS Genet. 2014 Jun 19;10(6):e1004411. doi: 10.1371/journal.pgen.1004411 (PMC4063673; doi:10.1371/journal.pgen.1004411)
Supplement: Table S2 — Plasmids used in this study. (DOC) [file pgen.1004411.s010.doc]

| Plasmid # | Name | Backbone | Type | Marker | Figure |
| --- | --- | --- | --- | --- | --- |
| 124 | pRS405-*pSGO1*-*SGO1*-TAP | pRS405 | Yeast integrative (YI) | *LEU2* | 1A, 1B, 1C, 1D |
| 176 | pRS405-*pSGO1*-*sgo1-*N51I-TAP | pRS405 | YI | *LEU2* | 1A, 1B, 1C, 1D |
| 127 | pRS405-*pSGO1*-*sgo1-*T379D-TAP | pRS405 | YI | *LEU2* | S1D |
| 279 | pRS405-*pMTW1*-*MTW1*-*RTS1* | pRS405 | YI | *LEU2* | 1C |
| 280 | pRS405-*pMTW1*-*MTW1*-*CDC55* | pRS405 | YI | *LEU2* | 1C |
| 216 | pRS405-*pMTW1*-*MTW1*-*SGO1*-eGFP | pRS405 | YI | *LEU2* | S1E |
| 218 | pRS405-*pMTW1*-*MTW1*-eGFP | pRS405 | YI | *LEU2* | S1E |
| 225 | pRS406-*pSGO1*-*sgo1-*N51I-*CDC55* | pRS405 | YI | *URA3* | S1F |
| 222 | pET-21b-*SGO1∆C* | pET-21b | bacterial expression plasmid |  | S1A, S1B |
| 224 | pET-21b-*SGO1∆C-*N51I | pET-21b | bacterial expression plasmid |  | S1A, S1B |
| BZ427 | pRS406-*pGAL1*-*cik1-cc*-TAP | pRS406 | YI | *URA3* | 1E, 2E |
| BZ454 | pRS406-*pGAL1*-*cik1-cc*-eGFP | pRS406 | YI | *URA3* | 2D |
| BZ8 | pRS425 | pRS425 | 2µ | *LEU2* | 5F |
| 365 | pRS425-*RTS1* | pRS425 | 2µ | *LEU2* | S3B |
| BZ40 | PRS425-*SLI15* | pRS425 | 2µ | *LEU2* | 5A–F |
| BZ10 | YEp24-*BIR1* | YEp24 | 2µ | *URA3* | 5A, B, E, F |
| BZ242 | pRS316-*SGO1* | pRS316 | CEN | *URA3* |  |
| 314 | pRS306-*pRTS1*-*RTS1*-TAP | pRS306 | YI | *URA3* | S2G |
| BZ372 | pRS406-*pGAL1*-*RTS1*-TAP | pRS406 | YI | *URA3* | S2F, S2G |
| BZ323 | pRS316-*pGAL1*-*CDC55*-TAP | pRS316 | CEN | *URA3* | S2F, S2G |
